# Identifying Frames of the COVID-19 Infodemic: Thematic Analysis of Misinformation Stories Across Media

**DOI:** 10.2196/33827

**Published:** 2022-04-13

**Authors:** Ehsan Mohammadi, Iman Tahamtan, Yazdan Mansourian, Holly Overton

**Affiliations:** 1 School of Information Sciences University of South Carolina Columbia, SC United States; 2 School of Information Sciences The University of Tennessee Knoxville, TN United States; 3 School of Information and Communication Studies Charles Sturt University Wagga Australia; 4 Bellisario College of Communications The Pennsylvania State University University Park, PA United States

**Keywords:** COVID-19, pandemic, misinformation, fake news, framing theory, social media, infodemic, thematic analysis, theme, social media, pattern, prevalence

## Abstract

**Background:**

The word “infodemic” refers to the deluge of false information about an event, and it is a global challenge for today’s society. The sheer volume of misinformation circulating during the COVID-19 pandemic has been harmful to people around the world. Therefore, it is important to study different aspects of misinformation related to the pandemic.

**Objective:**

This paper aimed to identify the main subthemes related to COVID-19 misinformation on various platforms, from traditional outlets to social media. This paper aimed to place these subthemes into categories, track the changes, and explore patterns in prevalence, over time, across different platforms and contexts.

**Methods:**

From a theoretical perspective, this research was rooted in framing theory; it also employed thematic analysis to identify the main themes and subthemes related to COVID-19 misinformation. The data were collected from 8 fact-checking websites that formed a sample of 127 pieces of false COVID-19 news published from January 1, 2020 to March 30, 2020.

**Results:**

The findings revealed 4 main themes (attribution, impact, protection and solutions, and politics) and 19 unique subthemes within those themes related to COVID-19 misinformation. Governmental and political organizations (institutional level) and administrators and politicians (individual level) were the 2 most frequent subthemes, followed by origination and source, home remedies, fake statistics, treatments, drugs, and pseudoscience, among others. Results indicate that the prevalence of misinformation subthemes had altered over time between January 2020 and March 2020. For instance, false stories about the origin and source of the virus were frequent initially (January). Misinformation regarding home remedies became a prominent subtheme in the middle (February), while false information related to government organizations and politicians became popular later (March). Although conspiracy theory web pages and social media outlets were the primary sources of misinformation, surprisingly, results revealed trusted platforms such as official government outlets and news organizations were also avenues for creating COVID-19 misinformation.

**Conclusions:**

The identified themes in this study reflect some of the information attitudes and behaviors, such as denial, uncertainty, consequences, and solution-seeking, that provided rich information grounds to create different types of misinformation during the COVID-19 pandemic. Some themes also indicate that the application of effective communication strategies and the creation of timely content were used to persuade human minds with false stories in different phases of the crisis. The findings of this study can be beneficial for communication officers, information professionals, and policy makers to combat misinformation in future global health crises or related events.

## Introduction

### Background

The contagious SARS-CoV-2 virus caused a global pandemic that has influenced many aspects of people’s lives across the world since early 2020. Due to the global scale of the pandemic, different stakeholders created and circulated an abundance of true and false information through various channels to fill the uncertainty in this crisis. Unfortunately, the sheer volume of false or fake information was such a severe problem during the pandemic that the World Health Organization (WHO) announced that battling misinformation was as challenging as fighting the virus itself [1]. Information disorder is defined with different terminologies such as misinformation, disinformation, and malinformation [2]. Disinformation is created to harm people with the aim of gaining money, political manipulation, and hurtful social and psychological consequences, while misinformation refers to sharing false information unintentionally [3]. The velocity of misinformation was one major issue to handle in the case of COVID-19. For instance, according to a Pew Research Center survey, around half (48%) of respondents had encountered false stories about the COVID-19 pandemic [4].

Therefore, research on false information specifically pertaining to COVID-19 is necessary because it will help to gain deeper insights into this issue and to manage similar crises more efficiently in the future. One main step toward this goal is to identify and classify COVID-19 misinformation stories that provide the necessary contextual data to understand the current ecosystem of unhealthy information. Several previous studies have reported COVID-19 misinformation, yet they are limited to a specific medium such as Facebook [5], Twitter [6,7], or YouTube [8]. Some earlier research includes only narrow samples [9,10] and theoretical frameworks [11,12]. Hence, it is important to explore the motivations and sources of false information and to discover its progress and prevalence on different platforms over time. Additionally, it is unclear how COVID-19 misinformation is framed and presented to the public on different platforms. Thus, this study targeted a comprehensive sample of false stories reported by fact-checking websites.

To fill these gaps in the literature, this study aimed to apply arguments from framing theory and thematic analysis to identify the main subthemes related to COVID-19 misinformation on a wide range of platforms from traditional outlets to social media. Another objective of this study was to explore the changes in the prevalence of misinformation subthemes over time.

### Literature Review

#### Theoretical Framework

Framing, as a concept, refers to attempts to include and highlight specific aspects of a reality related to a phenomenon while excluding or minimizing other elements of it [13]. Framing refers to selecting some aspects of an issue to make them more noticeable in communication [14]. Framing is useful at both the macro and micro levels. Macro-level framing emphasizes the reflection, motivations, and goals of message senders [14], while micro-level effects focus on the ways message receivers see, understand, and act on messages [15]. Framing theory has been applied in a wide range of academic fields such as psychology, sociology, communication studies, and information science [16].

Framing studies can be divided into 2 broad levels: content research and effects research. Content research aims to analyze the messages to identify and categorize existing frames, while effects research investigates the most influential frames to achieve a targeted result, such as changing the attitudes or behaviors of audiences [17]. Effects research also analyzes frames that exist within communicated messages (possibly identifying or categorizing frames).

Framing theory asserts that how messages are framed and presented to the public can have different impacts on public opinion, behavior, and actions. A slight change in how a message is framed can sometimes have a significant impact on public opinion [18]. For instance, Tahamtan et al [19] showed that people used various hashtags on Twitter to frame their opinion about COVID-19. They, for instance, showed that the “conflict” frame, despite its low frequency, had received high attention among Twitter users. Therefore, it is important to study how COVID-19 misinformation is framed and presented to the public. In this study, we used framing as a theoretical framework to investigate how misinformation about COVID-19 has been framed on various platforms.

#### COVID-19 and Misinformation Consequences

Recent studies on disseminating misinformation about the COVID-19 pandemic [20] through online media have illuminated both the means through which false information is spread and the implications that such information has on the public’s response to national and global health crises.

The dissemination of false health information, specifically on social media, can negatively affect peoples’ perceptions, beliefs, decisions, and actions [21]. For instance, past studies indicate that misinformation regarding vaccine safety can manipulate public opinion about vaccines and negatively affect immunization rates [22].

Through creating the dual-inheritance model of conspiracy theories, Mulukom [23] found that periods of public unease and uncertainty about public issues such as COVID-19 create conditions upon which those who are underinformed, lack trust, feel uncertain, and threatened are more likely to propel conspiracy theories. However, misinformation statements may receive different amounts of attention from the public. For instance, Enders et al [24] showed that general misinformation about COVID-19 received more attention than more specific misinformation such as the “the treatment and transmissibility” of COVID-19.

Right after the COVID-19 pandemic started in early 2020, due to public uncertainity about the virus and widespread misinformation, several studies investigated misinformation related to this health crisis from various perspectives. For instance, Flew [25] demonstrated that mistrusting the news could lead to a catastrophic societal unfolding during COVID-19. Laato et al [26] discovered that the higher degree to which someone trusts online media and information, the more likely they are to share unverified information about COVID-19.

A study by Li and Scott [27] investigated how fake news was spread after a well-known Chinese soccer player, Wu Lei, contracted COVID-19. According to this study, news (and consequently, fake news) about famous people tends to receive high attention from the public. This study found that social media such as Weibo and WeChat and self-media (ie, user-generated content) tend to worsen the spread of false information about COVID-19. Kouzy et al [28] also showed that COVID-19–related misinformation statements were mostly distributed by individual or group accounts and unverified Twitter accounts.

Past studies have investigated various aspects of COVID-19 misinformation across different countries. For instance, Kim et al [29] maintained that, in the early stages of COVID-19, being exposed to general information about the pandemic made people realize they need further information, while exposure to misinformation would make individuals realize they need to obtain less information about the pandemic, which consequently has negative consequences on people. This study also indicated that there are cultural differences in how people in different countries interpret and respond to misinformation during a global pandemic. Soto-Vásquez et al [30] studied the correction of misinformation regarding COVID-19 in families and communities in the United States and Mexico. The study found that, while there is a general reservation to dispel misinformation that appears on social media, family and friends are more likely to correct misinformation through text messages and everyday conversations. Through exploration of online religious misinformation in the Middle East and North Africa, Alimardani and Elswah [9] identified how new parameters for religion that have been created through the internet would create distinct regional and religious types of false information. Meese et al [31] investigated the deep-rooted societal unease with mobile infrastructure and technology and its connection to the rise in conspiracy theories that suggest COVID-19 and 5G are related in Australia, the United States, and the United Kingdom. Apuke and Omar [32] created a predictive model to determine that altruism, instant news sharing, socialization, and self-promotion are the main factors behind COVID-19 misinformation dissemination on social media in Nigeria. Notably, entertainment was not found to have any connection to the propagation of fake news about COVID-19 on social media.

The literature review showed that past studies on COVID-19 misinformation are limited to specific contexts such as religious misinformation [9], geographical areas [29], or platforms such as Twitter [7]. These studies may not represent all aspects of COVID-19 misinformation. Only one study has examined misinformation through fact-checking resources, but this study only used Spanish fact-checking resources [33]. Therefore, this study aimed to fill the gap in the literature by examining misinformation related to COVID-19 on various fact-checking websites.

#### Framing Misinformation on COVID-19

A few studies have investigated the framing of COVID-19 misinformation. For instance, by investigating 4 conspiracy theories about COVID-19, Bolsen et al [21] found that encountering fake messages about COVID-19 was detrimental to how the public had framed health messages; this could lead to this global pandemic not being taken seriously. Bolsen et al [21] indicated that exposure to framed messages regarding the origins of COVID-19 can have a powerful effect on people’s beliefs about the cause of the global pandemic. Moreover, beliefs about the origin of the virus had strong “downstream effects” on respondents’ willingness to penalize China when they believed it may have been created by the Chinese government. Conversely, results indicated that those who believed the virus originated naturally, from zoonotic transmission, were more supportive of additional funding for biomedical research to identify harmful coronaviruses. This study also indicated that exposure to a conspiracy theory about the virus’s origin, in isolation or in competition, resulted in a “conspiracy effect,” which led individuals to be less likely to view actions such as wearing face masks, washing hands, and maintaining social distancing as important for alleviating the effects of the pandemic [21].

Using framing analysis of misleading YouTube videos about COVID-19, Rooke [34] found that risk amplification for their online audiences was the main goal of far-right misleading information sources. Using a narrative research design, with in-depth interviews with 19 individuals in Western Kenya, Chamegere [35] investigated which misinformation and conspiracy theories about COVID-19 were rising in Kenya. He also examined how people framed their opinions about those conspiracy theories. Results indicated that people framed their opinions about COVID-19 misinformation as follows: COVID-19 is “no worse than normal flu,” “a biological weapon,” “a political tool theory,” “a religious conspiracy theory,” and “an isolation theory.” Brennen et al [36] examined the most common visual frames related to COVID-19 misinformation. They identified 6 frames, including authoritative agency (claims about actions of public authorities), intolerance (expressions of racism, xenophobia, and sexism), virulence (claims that the virus is not real), medical efficacy (claims that treatments exist for the virus), prophecy (claims that the virus has previously been predicted), and satire (humorous content).

This literature shows that, although some past studies have explored COVID-19 misinformation, how misinformation stories have been framed on different platforms and at different time periods is understudied. Only a few studies have reported results regarding how misinformation has been framed, but they are limited to specific areas or contexts such as the study by Chamegere [35] in Western Kenya. The current study fills the gap in the literature by studying the major misinformation stories that were covered by 6 fact-checking websites, meaning this study is not isolated to any specific area or context.

## Methods

### Data Collection and Analysis

Identifying misinformation on the social web is a challenge for researchers. For this study, false information cases that were reported by fact-checking websites were selected and analyzed. Fact checking refers to the process in which journalists, experts, and nonprofit organizations use different sources and methods to systematically evaluate the validity of a claim and examine whether it is factual [37]. This approach is less biased because fact-checking websites not only are maintained by professional journalists and experts but also use rigorous procedures to identify and report false and misleading information. In addition, these websites monitor traditional and social media platforms that cover diverse information channels where users get their daily information. Therefore, the quality and methods used to identify false stories in this study were checked by journalists and professionals, rather than by the authors of this paper. Between January 2020 and March 2020, 8 different fact-checking websites (listed in Table 1) were monitored, and 2 researchers checked these websites manually to find and extract COVID-19 misinformation stories. Finally, 127 pieces of false news related to COVID-19 were found and collected.

**Table 1:** List of fact-checking organizations that was used for data collection.

Name	Managed by	URLs
Factcheck	University of Pennsylvania	Factcheck.org
The Fact Checker	Washington Post	washingtonpost.com/news/fact-checker
Media Bias/Fact Check	Independent Media	mediabiasfactcheck.com
PolitiFact	Poynter Institute	politifact.com
Snopes	Snopes Media Group	snopes.com
TruthOrFiction	Whats True Incorporated	truthorfiction.com
RealClearPolitics	RealClear Media Group	realclearpolitics.com

In the next step, thematic analysis was applied to all 127 false stories. Thematic analysis was used because it helps to discover aspects, similarities, and differences within the false information stories [38]. Thematic analysis is a common methodology for identifying main themes in framing studies [17].

First, researchers read the full stories, multiple times and separately, to identify occurring patterns in the data sets. The original false news was referred to in order to maintain a better understanding of the data. A deductive approach was utilized in a meeting, and researchers brainstormed about the existing themes using available resources, mainly news and reports. In addition, inductive analysis was applied in this study. Each researcher individually developed their own themes with clear descriptions for each subtheme and theme by reading the misleading stories fully. NVivo, a qualitative data analysis tool, was used to sort, organize, manage, and analyze the qualitative data. Researchers reviewed the themes that they assigned to the data independently. Cohen kappa was used to evaluate intercoder reliability [39]. The agreements between 2 coders ranged from .8112 to 1 across all identified subtheme and themes. The Fleiss guidelines considers Cohen kappa values above .75 to indicate strong agreement levels. [40].

### Ethical Considerations

All data used in this project are secondary data from fact-checked websites that are accessible to the public on the web. This study did not use and analyze any personal or individual information.

## Results

### Themes and Subthemes

Following approaches from extant literature [41], the researchers first identified 4 main themes from the 127 pieces of news that were analyzed: attribution, impact, protection and solutions, and politics. Within these themes, 19 subthemes emerged. They are summarized in Table 2 and described in the following sections.

**Table 2:** Identified COVID-19 misinformation themes, subthemes, and frequencies in the studied sample.

Themes and subthemes		Examples	Frequency
**Attribution theme**			
	Origination and source	5G, lab-created	20
	Pseudoscience	Scientists believe; COVID-19 comes from bats; Charles Lieber.	11
	Origination date of the virus	Lysol knew; coronavirus is not actually new.	11
	Biological weapon and war	Virus was created as a bioweapon.	5
	Religious	Sent by God to punish homosexuals and environmentalists.	4
**Impact theme**			
	Fake statistics	65 million deaths	14
	Not severe and exaggerations	Media is exaggerating the risks of COVID-19; coronavirus is the least deadly virus.	9
	Racist issues	Africans are genetically resistant to coronavirus.	4
	Health costs	The United States is charging over $3.00 to test for COVID-19.	3
**Protection and solutions theme**			
	Travel and transportation	The United States would suspend “all travel from Europe” for the next 30 days, excluding the United Kingdom.	7
	Stopping or containing the virus spread	The number of COVID-19 cases in the United States, as of February 27, was decreasing.	6
	Quarantine	Trump will mandate 2-week in-home quarantine for the nation.	4
	Home remedies	Chlorine dioxide; vinegar, garlic water; warm water	16
	Treatments and drugs	Saline; hydroxychloroquine	12
	Diagnosis and testing	Hold your breath without coughing; diabetic monitors and complimentary testing kits for the coronavirus	7
	Virus killers	Virus is killed at 26/27 degrees.	5
	Personal protective equipment	Hand sanitizer will do nothing for the coronavirus; face masks should only be worn by medical professionals.	4
**Politics theme**			
	Governmental and political organizations	Democrat party, Chinese Communist Party; US Department of Homeland Security, Chinese Government; Spanish Army, US Army; CDC^a^	38
	Administrators and politicians	Donald Trump, Nancy Pelosi	24

^a^CDC: Centers for Disease Control and Prevention.

#### Attribution Theme

##### Origination and Source

Any inaccurate or unproven information related to the source of the virus was classified in this category. Some internet users blamed governments of some countries, such as Canada, China, and the United States, for producing the COVID-19 virus:

Canada is the source of the 2019 coronavirus outbreak in China.

The US was interested in the bioweapon and the deal to transfer the virus accidentally released it in Wuhan.

Government lab sent pathogens to the Wuhan facility prior to the coronavirus outbreak in China.

Another type of false information about the root of COVID-19 argues that the virus was created in a lab by humans:

There was an accidental leak of lab-created coronavirus.

The new coronavirus contains HIV insertions and shows signs of being created in a lab.

Certain products have also been stated to be the root of the virus. For instance, it was said that:

COVID-19 was found in toilet paper, and a strain of the dead virus breeds rapidly in tissue fiber.

The virus is an American product par excellence, according to the registry of inventions submitted in 2015.

Some other statements claim that famous figures are the root of the virus, such as professors or celebrities. For instance, it was stated that:

Harvard professor Charles Lieber has been arrested for creating the coronavirus.

A related misinformation story about artists claimed that:

Sam Hyde is responsible for the spread of the new coronavirus.

Other falsely claimed sources of COVID-19 are related to technology, such as 5G:

5G has damaged people’s immune systems.

##### Pseudoscience

Another type of misleading information pertained to unproven scientific facts and claims related to different aspects of COVID-19. Some argued that there are existing scientific solutions such as patents or medications for the virus:

There is a patent for the virus, and a vaccine is already available.

Some focused on the misinterpretation of scientific findings, for example:

Scientists believe that coronavirus may have come from bats in a Chinese research facility.

##### Origination Date of the Virus

There were some incorrect claims that COVID-19 was a known virus before 2019:

Clorox bottle claimed it could kill 2019 coronavirus before it was developed, proving that the virus was developed prior to the outbreak.

Lysol knew about coronavirus before it was common knowledge or spreading in humans.

Some of this false information argued that medications for the virus were available before the pandemic, for instance:

There is medication for the coronavirus that proves that the novel coronavirus is not actually new and has been known about for years.

Another example indicated that the Centers for Disease Control and Prevention (CDC) was aware of the virus:

The CDC had “advanced knowledge” of the COVID-19 outbreak in November 2019.

##### Biological Weapon and War

This category consists of statements that falsely claimed COVID-19 was created as a biological weapon by the Chinese or US governments to possibly pursue their political or economic goals against other countries. For instance, a false claim related to the United States was:

The coronavirus is part of the American biological war against Russia and China.

A spokesman for the Chinese foreign ministry claimed that the coronavirus did not originate in a Wuhan market, but rather was weaponized deliberately by US troops taking part in an athletic competition in that city last year.

Some statements, also related to the Chinese government, have shown:

A picture depicting a railroad tanker car with the ‘COVID19’ labeling indicating the transportation of the virus across the country.

Rumor claiming that the virus was created by the Chinese Government as a bioweapon to be released on the people of China.

##### Religious

Some misleading information about COVID-19 is related to several religious issues. Some of these stories focus on religious leaders. An example includes a fabricated story about Pope Francis:

Pope Francis and two of his aides have tested positive for the novel coronavirus.

Some piece of news connected the pandemic to Saint Corona:

Saint Corona is the patron saint of epidemics.

Another subtheme in this category was religious myths, such as:

Covid was sent by God to punish homosexuals and environmentalists.

#### Impact Theme

##### Fake Statistics

As shown in Table 2, some stories focused on fake predictions about various aspects of COVID-19. For instance, a piece of news claimed that:

Health experts predicted the new coronavirus could kill 65 million people.

Another example was the false news about the forecast done by Gates foundation:

The Gates foundation and others have predicted up to 65 million deaths from the coronavirus.

Additionally, some fabricated statistics circulating the internet referred to increasing and decreasing COVID-19 cases and deaths, such as:

The coronavirus will kill Ukraine in days, according to the expert Olyaksandr Teplyuk.

The number of COVID-19 cases in the US, as of Feb. 27, was decreasing.

##### Not Severe and Exaggerations

These statements claimed that COVID-19 and the pandemic are not as severe of a problem as others are claiming, for instance:

The coronavirus is the least deadly virus.

Novel coronavirus (COVID-19) is no more dangerous than the common cold.

Sweden declines treatment for coronavirus because virus is safe, and they have not closed borders.

Particularly, some stories claimed that consequences of the pandemic are not serious issues (including the economic impact and deaths). For instance, it was falsely claimed that:

The global economic impact of the shutdown – could be for nothing.

In terms of the global population, COVID-19 mortality figures are insignificant, and indicates natural process.

Some statements tried to provide evidence by citing sources such as a photograph that shows the role of media and journalists in exaggerating the risks of the virus, for example:

A photograph proves the media is exaggerating the risks of COVID-19 by showing a reporter in personal protective equipment.

##### Racist Issues

This category is about blaming the Chinese, as a nationality or ethnicity, for causing and spreading the COVID-19 virus. Some false statements attributed the root of the virus to the Chinese Communist Party, for instance:

The Chinese Communist Party will admit that there was an accidental leak of lab-created coronavirus.

Other false statements or claims in this category included:

The 1918 influenza pandemic was called the “Spanish Flu” because it emanated from Spain, so the Chinese should be fine with the US referring to COVID19 as the “Chinese virus” or “coronavirus may have come from bats in a Chinese research facility”.

##### Health Costs

This subtheme consists of false claims related to COVID-19 costs, such as the decision of authorities to waive copayments. For instance, it was claimed that:

Industry leaders agreed to waive all copayments.

This subtheme also contains information about the COVID-19 testing costs. For instance, it was stated that:

The US is charging over $3,000 to test for COVID-19.

Another example is a false claim noting that:

There are free diabetic monitors and complimentary testing kits for the coronavirus for diabetics using insulin.

#### Protection and Solutions Theme

##### Travel and Transportation

This category covers any false news related to human travel, as well as transportation and travel restrictions, and their consequences (see Table 2 for more information), for example:

The U.S. would suspend “all travel from Europe” for the next 30 days, excluding the U.K.

The positive or negative consequences of false claims about travel and restriction include impacting trade and cargo, saving lives, contracting the virus, and protecting populations, for instance:

The Coronavirus will be the end of globalization with states and countries closing borders in order to protect their population.

Another example is:

Wish.com ships all products from Wuhan, China, and Wish.com products might cause you to contract coronavirus.

##### Stopping or Containing the Virus Spread

This category consists of incorrect claims about stopping and decreasing the spread of viruses. Some statements falsely claimed that the virus has been contained, such as:

COVID-19 has been contained.

Chinese officials were seeking approval from the Supreme People’s Court to start the mass killing of 20,000 people infected with the coronavirus in an attempt to contain.

Some statements claimed that the number of COVID-19 cases is decreasing, such as: “The number of COVID-19 cases in the US, as of Feb. 27, was decreasing.”

Other false claims in this category were related to the actions taken by officials to prevent or slow down the spread of COVID-19. For instance, it was stated that:

Images show the Spanish Army in the process of locking the country down to prevent the spread of coronavirus strain COVID-19.

Belgium's health minister banned “non-essential sexual activities” in groups of three or more due to coronavirus.

Another example was related to the warm temperatures that would help to get rid of the virus, such as:

The coronavirus will go away in April, as temperatures warm.

##### Quarantine

This issue reflects misinformation related to all aspects of quarantine. There are some pieces of news about the “immediacy” of quarantine, for example:

A text message sent in mid-March from the White House stating there would be a national lockdown or quarantine within 48 hours.

Another aspect of focuses on the “mandatory” aspect of quarantine, for instance:

The Stafford Act, which will mandate a mandatory two-week in-home quarantine for the nation.

Additionally, this subtheme points to the consequences of the quarantine such as looting. For instance, it was claimed that:

There has been an increase in looting in San Francisco since the city entered a shelter-in-place order in March 2020.

##### Home Remedies

Home remedies include false and unproven information to cure or prevent COVID-19. The home remedies include drinking liquids such as garlic water, chlorine dioxide, and vinegar to kill the virus. It also included false information about the impact of hot air and water in killing the virus. For instance, it was claimed that:

Using a hair dryer to breathe in hot air can cure COVID-19 and stop its spread.

Gargling with saltwater or vinegar “eliminate” the COVID-19 coronavirus from the throat of an infected person's system.

##### Treatment and Drugs

This category includes issues related to false claims about the treatment of, and drugs used, to cure the COVID-19 disease. Some of the claims in this subtheme referred to the availability of immediate treatments for the disease. For example, a statement falsely claimed that:

There are two drugs, as of March 19, (chloroquine and remedesivir) that show promise as therapies for COVID-19 and have been approved and are available for immediate delivery.

Another aspect is related to the unproven claims about existing drugs used to treat COVID-19. For instance, some internet users shared that:

Russian doctors have found a way to treat the virus.3 drugs that are also used to fight HIV, Hepatitis C and MS (Multiple Sclerosis) are recommended.

Specifically, there were some stories referring to the use of traditional medicine in treating COVID-19, for example:

China was able to control the pandemic without a vaccine by using traditional and low-cost medicine.

##### Diagnosis and Testing

This subtheme includes incorrect information about the methods for the diagnosis of COVID-19 and misleading information about different aspects of testing. One aspect of this subtheme relates to methods for self-diagnosis and self-tests. One example for self-testing is:

If you can hold your breath without coughing, discomfort, stiffness, or tightness, your lungs do not suffer from fibrosis and therefore you have no COVID-19 infection.

Some false information focused on testing methods for specific diseases, for instance, “There are free diabetic monitors and complimentary testing kits for the coronavirus for diabetics using insulin.”

Another issue in this category relates to the availability of testing methods in the early days of the pandemic that claimed:

There is no shortage of coronavirus tests in the US.

Also, there was a false story that discusses the interference of politicians to make the testing more difficult:

The Obama administration officials made regulations that have made it difficult to make testing for the coronavirus available.

##### Virus Killers

This category includes false information about the ways the virus can be killed, including heat and saline. Some false claims argued that the virus is not heat resistant. For instance, it was stated that:

The virus is not heat-resistant and will be killed by a temperature of just 26/27 degrees.

Coronavirus dies at 26-27 degrees (Celsius). Spring heat will overcome the coronavirus, and you also need to often drink hot drinks and spend more time in the sun.

On the other hand, some stories claimed the opposite, such as:

The virus is heat resistant and will be killed by a temperature of just 26/27 degrees.

Some claims also referred to saline as a substance for killing the virus, such as:

Coronavirus can be killed in 4 days by using saline.

##### Personal Protective Equipment

This category includes false information about personal protective equipment such as masks and sanitizers. For example, it was claimed that:

Face masks should only be worn by medical professionals.

Another type of misleading information in this category is related to the ineffectiveness of washing hands, such as:

Hygienist criticizes measures to protect against COVID-19 and states “Washing your hands is useless.”

Hand sanitizer will do nothing for the coronavirus.

#### Politics Theme

##### Governmental and Political Organizations

This theme includes false information that internet users have created and disseminated about authorities at the organizational levels, including governments, governmental agencies, political parties, health care institutions, and military forces. The false information in this category contained rumors related to the role of governmental and political organizations about different aspects of COVID-19 such as economic impacts, the virus’ roots, and border crossings.

For instance, a false piece of information about the US Department of Homeland Security claimed:

The US Department of homeland security said that they fear illegal border crossings may increase the spread of the novel coronavirus.

Another example about the US government attempting to control economic impacts was indicated in a post that has garnered more than 5000 shares and stated that:

All US Citizens are Entitled to $700 USD per week to stay at home to avoid the spread of COVID-19 novel Coronavirus, starting from March 17, 2020.

Another post indicated:

The Government grant pay is accessible to all no matter employment status.

Some stories focused on political and governmental institutions as the root cause of the virus. As an example, social media users created a rumor “claiming that the virus was created by the Chinese Government.”

An example related to health organizations claimed “The CDC had ‘advanced knowledge’” of the COVID-19 outbreak in November 2019.”

Some false news was related to the engagement of military forces in creating the virus, for instance:

US military brought the virus to Wuhan.

Another aspect of this context regards using the power of the army as a strategy to control the pandemic, for example: “Images show the Spanish Army in the process of locking the country down to prevent the spread of coronavirus strain COVID-19.”

##### Administrators and Politicians

This category includes any rumor and misinformation related to administrators and politicians or rumors created by them at the individual level. These include politicians receiving personal benefits from the disease (eg, stock market manipulation) and politicians’ decisions about the virus (eg, travel restrictions, quarantine, regulations, funding the National Institutes of Health and CDC, national security council, scientists).

For instance, it was claimed that:

Nancy Pelosi was caught trying to include abortion funding in the bill to combat coronavirus.

Donald Trump owns stock in and stands to benefit from the use of testing machines produced by Thermo Fisher Scientific Corporation.

Another example in this category is the fake information created by Donald Trump that was published on the Web through his speeches and official Twitter account. For example, he claimed that:

Antiviral therapies will be available in no time.

This highlights his strategy to manage the pandemic in a short time. Another similar example is his claim about the effort of Google in developing an application for screening the virus:

Google is working on a screening website that large numbers of Americans can soon use to see if they should be tested for the coronavirus.

### Subthemes and Themes and Media Platforms

After completing thematic analysis, the platform(s) from which the stories had originated were re-checked to identify on which platforms each piece of news was primarily shared. In some cases, stories started from different platforms at the same time; when this occurred, more than one media platform was coded for these cases. It is possible that a story started in one medium and spread across others later, but we only considered the platforms on which the piece of news originated because it was difficult to track secondary media dissemination. Through checking the reported articles in the fact-checking websites, we identified the key platforms. The stories are mainly shared through websites such as *InfoWars* that are maintained by conspiracy theorists (n=79). Facebook was the second platform on which misleading information was created (n=69). In our sample, Twitter was the third-leading avenue by which people created misleading information (n=40). Another place where misinformation stories originated was mainstream media (n=18). This included some tabloid outlets and some official news agencies such as *Newsweek*, *CNBC*, and *Yahoo! News.* Other sources of misleading information were official government avenues, such as formal websites, press conferences, and briefings. White House channels were one of the examples for this category (n=18).

Due to the low frequency of YouTube, instant messaging, and Reddit in our sample, we merged them into a category labeled as *other* social media (n=5).

**Figure 1:**
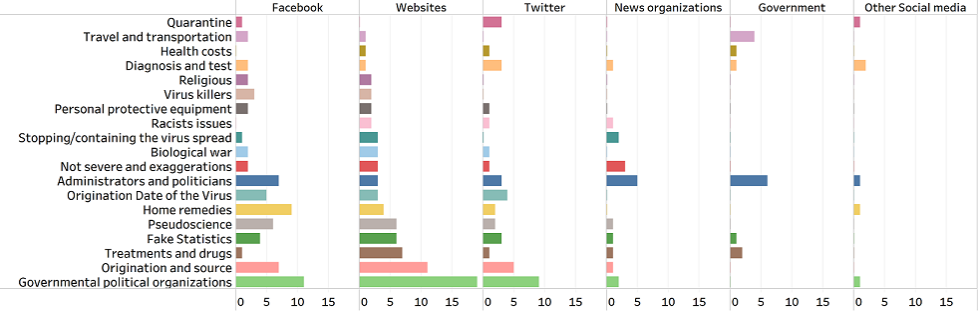
Distribution of COVID-19 misinformation frames across different platforms.

As Figure 1 shows, the frequency of misinformation has differed across platforms. “Governmental and political organizations” (9/40, 23%) and “Origination and source” (5/40, 13%) were 2 subthemes with high frequency on Twitter and websites (19/79, 24% and 11/79, 14%, respectively). “Administrators and politicians” was the popular subtheme on Facebook (7/69, 10%), mainstream media (6/18, 33%), and governmental outlets (6/15, 40%). “Home remedies” (9/69, 13%), “Travel and transportation” (4/15, 27%), and “Not severe and exaggerations” (3/18, 17%) were the second most popular subthemes on Facebook, governmental sources, and mainstream media, respectively.

### Subthemes and Time

**Figure 2:**
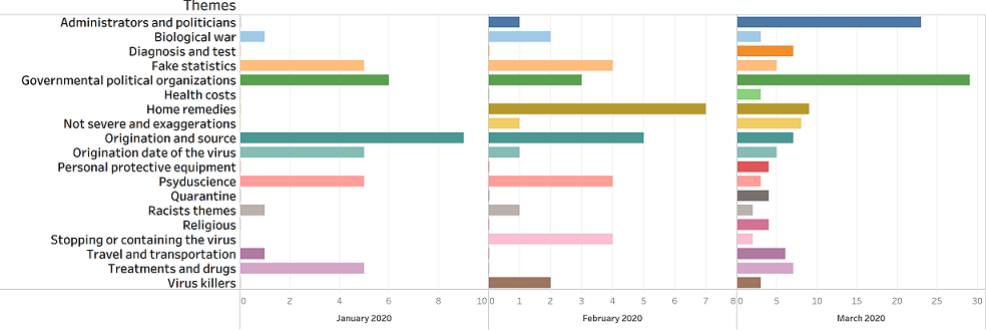
The prevalence of COVID-19 misinformation between January 2020 and March 2020.

This section explains the frequency of subthemes over time, from January 1, 2020 to March 30, 2020. Each month was split into 3 periods. Figure 2 shows that the subthemes were mentioned only 39 times in January, 35 times in February, and 135 times in March. Mid-March had the highest frequency (79/207, 38.2%) followed by late January (37/207, 17.9%) and early March (33/207, 15.9%). The high frequency of false information in March, specifically mid-March, is possibly because the COVID-19 disease was declared as a pandemic by the WHO on March 11, 2020 [42].

“Origination and source” (9/38, 24%) and “Governmental and political organizations” (6/38, 16%) were the 2 subthemes with the highest frequency in January. In February, again, “Origination and source” had the highest frequency (5/35, 14%), followed by “Fake statistics,” “Pseudoscience,” and “Stopping or containing the virus spread,” each with a frequency of 4 (4/35, 11%). In March, “Governmental and political organizations” (29/134, 21.6%), “Administrators and politicians” (23/134, 17.2%), and “Home remedies” (7/134, 5.2%) had the highest frequencies.

Most of the subthemes, such as “Administrators and politicians” and “Home remedies,” had increased from January 2020 to March 2020. However, a few subthemes, such as “Treatments and drugs” and “Origination date of the virus,” experienced a decrease in February. For instance, “Treatments and drugs” was mentioned 5 times in January, decreasing to 0 in February, then increasing to 7 in March. The frequency of “Administrators and politics” was 0 in January, increasing slightly to 1 in February, with a significant increase to 23 in March.

## Discussion

### Principal Findings

Misinformation and disinformation are extremely complex and contextual concepts with various layers and levels. Therefore, it is not easy for people to distinguish credible information from fake or false news, especially in the case of an overly complicated crisis, such as the ongoing global pandemic. Even for people with information literacy skills, it is still not an easy task to avoid misinformation and disinformation, as the complexity of this issue is increasing constantly. Developing a more widespread awareness of influential misinformation categories could help people to be more informed and prepared when facing misinformation.

For example, as Table 2 indicates, “Government organizations” and “Administrators and politicians” were the top 2 subthemes in the results, which aligns with findings from a previous study [12]. There are 3 probable reasons for this. First, any news about this group will attract more attention from users as this group’s decisions have a tremendous impact on society. Second, it is extremely difficult for ordinary people to directly access this group to verify the validity of the information. Therefore, there is a paradoxical circumstance here: “Governmental and political organizations” and “Administrators and politicians” are more visible and less accessible at the same time. This paradox creates a fertile ground to produce misinformation related to this group more than ever. Finally, they are easy targets to blame for their poor decision-making and their incapability to deal with the crisis.

The third most frequent subtheme was “Origination and source” of the virus. This subtheme’s high frequency comes from the fact that any information about the origin of an unknown phenomenon has a higher chance of attracting peoples’ attention. Knowledge regarding a certain phenomenon helps people to lower their levels of uncertainty. In the case of the current pandemic, the level of uncertainty about the origin of the virus is extremely high for everyone, even for experts, and people seek explanations about the mysterious source of this unknown disease. They need to make sense of what is going on around the world, and any information about the source can lower their uncertainty level. Therefore, information on this aspect of the pandemic, regardless of its credibility and validity, will naturally be incredibly interesting for most people, and they pay consequently more attention to it. With consideration to media framing, these subthemes are indicative of information sources “declaring the underlying causes and likely consequences,” as suggested by extant research [43]. This is also an exemplification of early arguments by Iyengar and Simo [44] about attributing blame for a societal issue.

The fourth category was “Home remedies,” probably because, during this disastrous time, people desperately seek solutions, especially easy solutions, and home remedies sound like promising options for many. Therefore, any information about home remedies has a higher level of attractiveness and will inevitably create another rich ground for misinformation creation and dissemination. Here, a distinction between thematic versus episodic framing emerges, as many of the shared home remedies are a result of a specific case or example, or episodic framing [45].

The fifth category was “Fake statistics.” The reason behind its popularity is the power of statistics to more precisely and correctly show false stories. This communication strategy helps to represent incorrect information more evidentially and to persuade minds as a result [46]. The capacity of figures to exaggerate any situation may be another reason for the creation of fake statistics. For example, the number of people affected by the disease, or the economic consequences of the pandemic, can be easily summarized into statistics that cannot be verified by people; however, it can attract attention. Moreover, browsing numbers and figures is often much easier for people than reading long stories.

The next 4 categories, including “Treatment and drugs,” “Pseudoscience,” “Not severe and exaggerations,” and “Origination date of the virus,” have a shared element that can potentially accelerate the dissemination of misinformation. The shared element among these 4 categories is a form of denial for people, that the new disease is not a big problem and there is nothing serious about it. One of the reasons for this denial is related to an orchestrated strategy to show that organizations and decision-makers are not responsible for managing crises, and it is a known application of false information in crisis communication [47]. Another aspect of the denial is pertinent to the abuse and misinterpretation of research and scientific discoveries. This can be another tactic of misinformation to manipulate public opinion, which has been reported in previous research about misinformation and climate change [48]. These types of misinformation may attract attention because people are looking for relief and comfort in crisis, and this kind of news will be very appealing; thus, they pay more attention to it.

In the quarantine subtheme, the claims were not false after March 15, 2020 when the WHO declared the disease as a pandemic and countries opted for mandatory lockdowns. This shows that a false claim may not be false anymore at another time. Context matters in discussing false information.

When considering the broader themes under which each of the above subthemes were classified, this study found that the “Protections and solutions” theme included the largest number of subthemes (8 subthemes), followed by the “Attribution” theme with 5 subthemes. The “Impact” theme included 4 subthemes, and the “Politics” theme included only 2 subthemes. Therefore, although the subthemes that reoccurred the most (government and political organizations as well as administrators and politicians) were within the “politics” theme, the theme with the largest number of subthemes was related to how individuals and our society can find solutions related to the pandemic.

Results of this study revealed the role of different platforms in circulating misinformation. Findings show that “hoax or conspiracy theorist news websites” were the primary sources of creating false information about COVID-19. This agrees with the results of a similar study about a specific false story [10,41]. In our sample, Facebook and Twitter were the 2 main social media sources of misinformation, which aligns with quantitative studies about the sources of false information about COVID-19 and previous health crises. Surprisingly, this study shows that trusted media, such as news agencies and official government platforms, were also sources of false stories in the pandemic, which is in line with a survey study in different countries [49].

The COVID-19 pandemic had different stages based on which misinformation subthemes were prevalent at the time. For instance, before March 11, 2020, it was known as an epidemic, while on March 11, 2020, it was declared as a pandemic by the WHO. At this stage, the globe experienced new challenges such as the mask mandate, quarantine, and panic about the shortage of products [19,50]. The type of misinformation could vary by the different stages of a pandemic. For instance, during the initial phase of a pandemic, when there is a lack of trust between politicians and the public [51] and there are high levels of uncertainty in society about the origins of the virus, nonverified information about the origins of the virus is more likely to be disseminated and possibly adopted by the public.

The results of this study indicate that “Origination and source” of the virus was one of the prevalent subthemes in the early phase of the pandemic, which is not surprising because right after the pandemic started, people around the world started exploring to learn more about the origins and causes of the virus. During this time, conspiracy theorists were rapidly spreading their ideas on social media, marketing their thoughts to the public, and shaping public opinion. “Origination and source” was still popular in February. These findings further support the findings of the study by Evanega et al [52]. Some conspiracy theories related to “Origination and source” were as follows: the relationship between 5G technologies and COVID-19, Gates’ plan to develop a vaccine using microchips, and bat soup as a source of the virus.

In February, “Home remedies” became a prominent subtheme for creating misinformation stories as COVID-19 went to another phase, that is, the public started taking it more seriously. As a result, people were searching for easy ways to cure the disease. In March, “Governmental and political organizations,” “Administrators and politicians,” and “Home remedies” were among the popular topics. These subthemes became more important because the actions and policies of governmental organizations to manage the pandemic were increasingly important to the public, and misinformation in these areas could attract more attention. In this period, the US presidential election was approaching, and people were more interested in information around political parties and COVID-19 issues that created a situation for misinformation. Additionally, as mentioned, March 11, 2020 was when the WHO declared the COVID-19 disease a global pandemic [42]. These subthemes had a common point, indicating that politicians tried to offer immediate and unproven solutions to stop, cure, or kill the virus. For example, the former president of the United States talked about hydroxychloroquine and chloroquine as treatments of COVID-19 on March 19, 2021, while there was no scientific evidence to prove this claim.

In summary, from a framing perspective, the results clearly suggest that there is a concerning amount of inaccurate information being disseminated across a variety of platforms concerning COVID-19. Results from this study clearly support the framing theory’s arguments about message themes and public opinion, as argued in previous research [18]. For example, the “Governmental and political organizations” subtheme that emerged as the top subtheme is reflective of a society that distrusts science and those in positions who strive for truth-telling in an era of misinformation, such as the CDC, Dr. Anthony Fauci, and others. Specifically, the findings identified that the analyzed stories most frequently included misinformation about politics. The “Origination and source” subtheme raises questions about attribution of responsibility. In a different context, scholars have argued that a correct understanding of the cause of an issue is the key to success in promoting mitigative behaviors [53]. The false information identified across several subthemes in this study raises concern about individuals and their interest in, or ability to, act responsibly during the pandemic because of a lack of factual information. Subthemes such as “Fake statistics” and “Origination date of the virus” present information in a way that might diminish individuals’ willingness to engage in responsible behaviors to combat the virus, which is also reflective of findings in unrelated framing studies that examined how message themes impact public opinion, behaviors, and actions [54-56]. These are important considerations as we aim to inform and educate individuals, and we continue to combat misinformation that can have detrimental effects on health and society.

### Conclusions

This study identified a wide range of subthemes and elements that are potentially significant for better understanding of information behavior patterns in this context (ie, pandemics). This study discovered that misinformation about authorities at the “organizational levels” (ie, rumors about the role of governmental and political organizations in issues such as economic impact and the source of the virus) and misinformation related to (or created by) administrators and politicians at the “individual levels” (ie, politicians receiving personal benefits from the disease) were more frequent than other types of misinformation.

The results also indicated that misinformation type and prevalence could vary by the different stages of a pandemic over time. These results could provide some insights for policy makers as well as communication and information officers to gain a better understanding of different phases of a crisis and take appropriate and timely actions. The actions could involve combating misinformation and designing better strategies to create correct content beforehand to help the public. Effective policies and practices focusing on this aim can minimize the harmful effects of this phenomenon. A global movement with local initiatives is necessary to increase public awareness of this problem and educate more people across the world in information literacy. Policymakers should engage in more evidence-based decision-making practices. Also, information service providers should offer more effective tools and techniques for their users to evaluate the authenticity and credibility of information sources.

Misinformation type and prevalence could vary by different platforms. This study confirms that web and social media platforms are the primary sources of misinformation, which is not unexpected. Surprisingly, though, results revealed trusted outlets of information such as government channels and known news agencies were platforms for creating COVID-19 misinformation as well.

In summary, regardless of its name, whether it is called disinformation, misinformation, fake news, or malinformation, this phenomenon is a form of “information disorder” and is a major threat to the global information landscape. It is a complex phenomenon, and there is no single way to fight it.

### Practical Implications

The catastrophic consequences of misinformation and disinformation on people’s lives are more disastrous than ever, especially during the current COVID-19 pandemic. The global crisis is much vaster than a smaller-scale health crisis and has numerous economic, social, and environmental aspects. Therefore, the results of this study can potentially present a range of practical implications for both policy makers and practitioners. At the policy level, policy makers can use the results to develop more effective policies to support dissemination of more trustworthy sources of information in society. At the practice level, practitioners can use the results to provide more effective and reliable services. For example, information professionals across the GLAM (Galleries, Libraries, Archives, and Museums) sector can identify the areas they need to focus on to enhance public awareness about the necessity of access to credible information in dealing with a challenging time like a global pandemic. Moreover, they can provide wider and more accessible learning opportunities for the public to empower people with higher levels of information literacy and media literacy skills. Furthermore, information system designers can use the results to identify the areas that require increased focus to help users find the most authentic and trustworthy sources of information. In addition, as this study found that web and social media platforms are the primary sources of misinformation, it is increasingly important for such platforms to issue information dispute warnings by flagging information that may be questionable or inaccurate. Finally, as individuals, members of society need to be vigilant and act as responsible media consumers to the best of their abilities. Until changes are incorporated at both the societal and individual level, there exists a risk of perpetuating the “information disorder” that has increasingly threatened the global information landscape.

### Limitations and Future Directions

This study has some limitations that should be noted. There are different private challenges such as closed Facebook pages, instant messaging applications, and emails that misinformation created and circulated. However, the content of these channels is not accessible for the fact-checking organizations to monitor systemically and, thus, are not part of the studied sample in this paper. Additionally, the time frame of this study was limited to a 3-month window, and it may not reflect the entire picture of false stories about COVID-19. Although fact-checking organizations aim to help provide factual data about misinformation in different contexts, they have some biases [57,58].

Further research is required to explore and reflect on each element with more qualitative and interpretive approaches. For example, conducting qualitative studies on these elements enables us to understand the actual impact of misinformation and disinformation on various aspects of everyday life during the pandemic. For instance, it can be explored to what extent dissemination of misinformation about the COVID-19 vaccination caused hesitation for various groups of people to delay their vaccination, and how this dilemma affected their real lives. In other words, what we need in further studies is a sample of real stories of real people to understand the actual influence of misinformation on various aspects of their life, ranging from their personal health and well-being to their financial and family issues. These real stories will shed light on some of the less-explored aspects of the damaging impacts of misinformation on people. Finally, some categories of misinformation could have a higher level of influence or impact on public perception, which should be investigated in future studies.

## Abbreviations

CDC: Centers for Disease Control and Prevention
GLAM: Galleries, Libraries, Archives, and Museums
WHO: World Health Organization
